# Combined BPA and DIBP Exposure Induced Intestinal Mucosal Barrier Impairment Through the Notch Pathway and Gut Microbiota Dysbiosis in Mice

**DOI:** 10.3390/foods14020214

**Published:** 2025-01-12

**Authors:** Mengge Duan, Yuting Wang, Shiyu Chen, Jiawen Lu, Ruihong Dong, Qiang Yu, Jianhua Xie, Yi Chen

**Affiliations:** State Key Laboratory of Food Science and Resources, Nanchang University, Nanchang 330047, China

**Keywords:** BPA, DIBP, intestinal mucosal barrier, notch pathway, gut microbiota, *Rubus chingii* Hu phenolic extract

## Abstract

Bisphenol A (BPA) and diisobutyl (DIBP) phthalate are widely used as typical plasticizers in food packaging. Plasticizers can be released from polymers, migrate into food, and be ingested by humans, leading to various health problems. However, little research has investigated the combined toxicity of BPA and DIBP, particularly their intestinal toxicity. Our goal is to analyse the combined toxicity of BPA (50 mg/kg) and DIBP (500 mg/kg) on the intestines of KM mice. Additionally, we tried to find natural products that can inhibit or prevent the combined toxicity of BPA and DIBP. The results indicated that the combination of BPA and DIBP exposure resulted in a reduction of beneficial flora, an increase in D-Lac levels (136 ± 14 μmol/L), an increase in intestinal permeability, activation of the notch pathway, and a decline in intestinal stem cells (ISCs) to goblet cells, compared to single-exposure sources. Nevertheless, *Rubus chingii* Hu phenolic extract (RHPE) (200, 400 and 600 mg/kg) ameliorated the BPA and DIBP-induced intestinal microbiota disruption and intestinal mucosal barrier impairment by inhibiting the overactivation of the notch pathway. The results of this study highlight the potential risks to human health posed by the combination of BPA and DIBP and may help explain the potential pathways of enterotoxicity caused by combined ingestion.

## 1. Introduction

Plasticizers are frequently employed in food packaging, including cling film, lunchboxes, soft packaging bags, and bottle caps [1]. Bisphenol A (BPA) and Diisobutyl phthalate (DIBP) are widely used plasticizers. The European Commission (EC) has stipulated that the concentration of DIBP in plastic toys intended for children should not exceed 0.1%. Exposure to PAEs in the population of different countries showed that the exposure level of DIBP in Kuwaiti residents (13.7 μg/kg bw/day) was significantly higher than that in residents of other countries such as the United States and Canada [2]. A study of developmental toxicity in SD rats revealed that at doses exceeding 500 mg/kg, female rats exhibited signs of developmental toxicity, including a decrease in body weight gain [3]. Nevertheless, the majority of research on DIBP has focused on reproductive and endocrine effects, with limited research on gut health [4].

The levels of bisphenol A (BPA) in food and food-contact materials are of utmost concern [5]. In April 2023, the European Food Safety Authority (EFSA) reduced the Tolerable Daily Intake (TDI) to 0.2 ng/kg bw/day, proposing a ban on the use of BPA in food-contact materials [6]. A study conducted in the United States determined that the average BPA concentration was 0.852 ng/g (meat and meat products), 3.23 ng/g (fish and seafood), 8.99 ng/g (vegetables, including canned vegetables), and 1.90 ng/g (fats and oils) [7]. Adults are exposed to 0.129 µg/kg bw/day on average, with the greatest exposure to BPA being 0.362 µg/kg bw/day [8]. Unfortunately, individuals are at risk for BPA exposure, and studies have found that BPA can be detected in peripheral blood, umbilical cord blood, amniotic fluid, follicular fluid, and urine [9]. In real-life scenarios, particularly in the context of food contact materials and pharmaceuticals, where multiple plasticizers have been used in combination, the assessment of exposure to a single contaminant does not accurately reflect the current state of exposure. Currently, the toxicity studies of plasticizers are limited to individual exposures, while the toxicity studies of combined exposures are lacking. As a result, the presence of compound effects was deemed a crucial factor in the risk assessment of plasticizers [10,11,12]. BPA’s reproductive toxicity in mice and rats was reported to have a NOAEL of 50 mg/kg/d [8]. Consequently, we selected BPA (50 mg/kg) [13] and DIBP (500 mg/kg) based on previous references [4,8,14,15,16].

N-acetylcysteine (NAC) serves as a potent antioxidant and plays a crucial role in regulating intestinal microecology in mice. The study demonstrated that NAC (2 g/L) significantly ameliorated obesity, dyslipidemia, and gut microbiota disruption induced by a high-fat diet (HFD) in mice [17]. NAC is employed as a feed additive to alleviate LPS-induced intestinal dysfunction through modulation of intestinal inflammation and permeability [18]. The “healthy composition” of the intestinal flora serves as a physical barrier against infections, whereas disturbances in the ecological balance of the intestine increase the susceptibility to pathogens [19]. Previous studies have indicated that exposure to plasticizers disrupts the homeostasis of the intestinal microbiota, characterized by an increase in the abundance of pathogenic bacteria such as *Muribaculum* and a decrease in the abundance of beneficial bacteria such as *Lactobacillus* [14,20]. Therefore, maintaining the homeostasis of the intestinal flora is a promising approach to mitigating the effects of plasticizers on intestinal damage. It was estimated that the dietary intake of polyphenols is largely unabsorbed in the small intestine and can accumulate in the large intestine, thereby extensively metabolizing the gut microbiota [21]. Consequently, polyphenols enhance the intestinal environment through interaction with the intestinal microbiota.

Polyphenol may enhance antimicrobial and anti-inflammatory properties, limit oxidation, lower blood sugar and cholesterol, and postpone aging [22]. *Forsythia suspensa* polyphenols regulate intestinal homeostasis in UC mice through the improvement of the intestinal flora [23]. The administration of an extract of *Broussonetia papyrifera* leaves (BPE, 200 mg/kg) significantly increased the abundance of commensal beneficial bacteria, such as *Faecalibaculum* and *Akkermansia genera* [24]. Our previous study showed that the main active ingredients of *Rubus chingii* Hu phenolic extract (RHPE) are tiliroside, kaempferol-3-O-rutinoside, ellagic acid and rutin. RHPE has the potential to effectively mitigate the damage caused by hydrogen peroxide in RAW264.7 cells [25]. The extent to which RHPE exerts a broad bactericidal and antioxidant effect as a palliative effect in intestinal injury induced by the combination of bisphenol a and DIBP remains unclear.

In addition to interacting with microorganisms, polyphenols play a multifaceted role in the treatment of intestinal injuries. Mucin-2 (Muc2) is the predominant secreted mucus protein. It was observed that muc2-deficient mice exhibit a looser, thinner mucus layer that facilitates bacterial penetration into epithelial cells [26]. Hence, we hypothesized that RHPE could enhance the intestinal mucosal barrier through stimulation of mucus secretion. HT29-MTX belongs to a subgroup of cup cells with mucus secretion. Caco-2/HT29-MTX co-cultured cells exhibited strong polarity, tight junctions, a thick mucus layer, and permeability values that were close to those of the human intestine [27,28,29]. Studies have demonstrated that cup cells are capable of responding to localized stimuli and challenges [30]. By limiting the transfer of trans-epithelial antigens to the immune system and strengthening the mucus barrier when it is disrupted, goblet cells in mice help protect the stomach from the microbiota. It was proposed that e-calmodulin enables bacteria to access previously inaccessible proteins, specifically to invade goblet cells [31]. Alterations in the intestinal mucus layer are associated with reduced numbers and/or impaired function of goblet cells [32]. The combined toxicity of BPA and DIBP in the Caco-2/HT29-MTX co-culture model was examined based on the CI. Two plasticizers’ combined effects on organisms are frequently categorized as additive, antagonistic, or synergistic effects, which are categorized as synergistic, additive, and antagonistic effects when CI < 1, =1 and >1, respectively [33].

The primary objective of this study was to conduct a preliminary investigation into the effects of BPA and DIBP on the gut microbiota. By doing so, we sought to determine if the mechanism of the combined exposure to BPA and DIBP effect on the intestinal microbiota was related to antimicrobial effects or secondary to digestive toxicity and the release of antimicrobial peptides. We conducted a preliminary investigation to determine if RHPE could mitigate the damage to the gut microbiota caused by combined exposure to plasticizers by exerting antimicrobial and antioxidant effects.

## 2. Materials and Methods

### 2.1. Chemicals and Reagents

We bought dimethyl sulfoxide (DMSO) from Sigma-Aldrich (St. Louis, MO, USA). BPA (purit > 99.0%), DIBP (purit > 99%), corn oil and N-acetyl-L-cysteine (NAC) from Aladdin (Shanghai, China). FITC-Dextran 4 kDa (FD-4) and berberine hydrochloride (BBR) were obtained from Solarbio Co., Ltd., (Beijing, China). Antibodies against Notch receptor 1 (Notch1), Delta-like 4 (DLL4), Hairy/enhancer of split 1 (Hes1), Mouse atonal homolog 1 (Math1), Mucin 2 (Muc2), and their corresponding secondary antibodies were obtained from Beyotime (Shanghai, China).

The species *Rubus chingii* Hu was collected from the planting base of Dexing (Jiangxi, China), and referring to the extraction method of the subject [25]. In our previous study, the main constituents of RHPE were quantified using HPLC-ESI-QqQ-MS/MS. The RHPE primarily consists of phenolic acid derivatives, flavonoids, and anthocyanins. Further quantitative analysis, the contents of kaempferol-3-O-rutinoside (495.88 μg/g dw), lindenoside (485.40 μg/g dw), rutin (495.88 μg/g dw), and ellagic acid (279.74 μg/g dw) were higher than the other compounds, accounting for approximately 96.63% of the total phenols. The results indicate that these four phenolic compounds may be the predominant components in RHPE.

### 2.2. Animal Experiments

**Figure 1:**
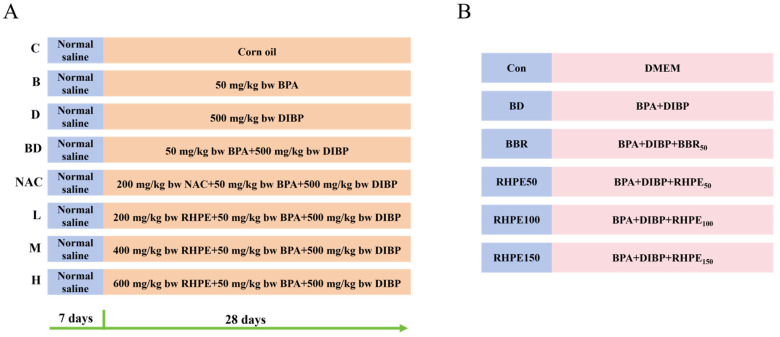
Experimental grouping scheme (**A**) Schematic diagram of the animal experiment. (**B**) Cell grouping.

Four-week-old specific pathogen-free (SPF) male KM mice were from Sipeifu (Co., Ltd., Beijing, China). The experimental mice were allowed to drink in a 12/12 h light/dark cycle animal home [34]. After a week rest period, these mice were randomly divided into Eight groups (*n*= 8 mice/group): the control group (C); BPA group (B); DIBP group (D); BPA + DIBP group (BD); BPA + DIBP + NAC group (NAC, as positive control); BPA + DIBP + RHPE200 group (L); BPA + DIBP + RHPE400 group (M); BPA + DIBP + RHPE600 group (H). The concentration of BPA is 50 mg/kg, DIBP 500 mg/kg, RHPE 200, 400 and 600 mg/kg, respectively. The detailed procedures are presented in Figure 1A. To summarize, mice were administered saline from day 1 to day 7, followed by corn oil, BPA, and DIBP on days 8 to 28, respectively. The NAC group and RHPE-pretreated group (L, M, H) were administered NAC and RHPE first, followed by BPA and DIBP (Table 1). The mice were sacrificed after anesthesia (CO_2_ inhalationc, 1–2 min) on day 28, and the samples were collected for the determination. After surgical resection, the liver and spleen were weighed separately, and the organ index was determined using the following formula:

Organ index (g/100 g) = Organ weight/body weight
(1)

All animal experiments were strictly carried out according to the Laboratory Animal Standards of Welfare and Ethics and were approved by the Animal Care and Use Committee of Nanchang University (Animal Ethics Number: NCULAE-20221030003).

### 2.3. Determination of Intestinal Permeability

D-lactic acid (D-Lac) levels in mouse serum were used to indicate intestinal permeability. D-Lac levels in serum were determined via the ELISA kit method according to the instructions of the reagent manufacturer (COIBO BIO, Shanghai, China).

### 2.4. Histopathological Observation

The prior method involved taking 2 cm of ileum tissue, fixing it with 4% paraformaldehyde, washing it with tap water, rehydrating it with alcohol, replacing it with xylenes, and finally encasing it in paraffin wax [35]. The encapsulated samples were then sectioned and stained with hematoxylin and eosin (H&E) and Alcian blue/periodic acids-Schiff (AB–PAS). A pathology scanner (Aperio LV1, Leica, Wetzlar, Germany) was used to visualize morphological differences in the ileum tissues. Using Image J 1.54 f (Silver Springs, MD, USA), measure the depth of the crypts, the length of intestinal villi, and the number of goblet cells.

### 2.5. Immunohistochemical Analysis

The ileum tissues were blocked and incubated for 1 h. Using primary antibodies anti-Muc2, anti-Lgr5, anti-Ki67, and anti-E-cad (Servicebio, Wuhan, China) at 4 °C overnight incubation. Then treated for 50 min with corresponding secondary antibody, followed by diaminobenzidine (DAB). Positive expression was visualized with the pathology scanner [36].

### 2.6. Gut Microbiota

Six randomly selected colon contents from each group were analyzed. The genomic DNA of fecal samples was extracted by using QIAamp^®^ Fast DNA Stool Mini Kit (Beijing, China). The primers were amplified to obtain the V3–V4 region of the bacterial 16S rRNA gene. PCR products were subjected to high-throughput sequencing using the Illumina platform, with data preprocessing including quality filtering, denoising, splicing, and dechimerization. QIME 2 (https://qime2.org, accessed on 14 April 2023) combines high-quality tags into operational taxonomic units (OTUs) at a 97% similarity criterion [37].

### 2.7. Determination of Contents of Short-Chain Fatty Acids (SCFAs)

The SCFAs contents were analysed following a previously reported method [38]. And 50–100 mg sample should be dissolved, homogenized, and centrifuged for 5 min at 13,000 rpm gathered the supernatant. Following filtration over a 0.22 μm sterile membrane, 0.2 mL 10% sulfuric acid and 0.5 mL anhydrous were added. Centrifuge at 13,000 rpm for 2 min, passing the supernatant through organic membrane then subjected for SCFAs analysis with 6890 N GC system (Agilent, Santa Clara, CA, USA).

### 2.8. Cell Culture and Solution Preparation

The Caco-2 cells were purchased from the Chinese Academy of. HT29-MTX cells were supplied by UP-Style Lab (Nanchang, China). Based on previous laboratory studies, we established a Caco-2/HT29-MTX co-culture model with a 90:10 ratio [39]. RHPE was added into DMEM to obtain the solution with different RHPE concentrations (0–200 μg/mL). The stock suspensions of BPA (60 mmol/L) and DIBP (400 mmol/L) were prepared in DMSO and diluted with DMEM to various concentrations, with DMSO below 0.05% during the entire assay.

### 2.9. Cell Viability

We evaluate the cytotoxicity of BPA, DIBP and RHPE by cell counting kit-8 (CCK-8 kits, APExBIO, Llc, Houston, Texas, USA). Co-cultural cells were inoculated in 96-well plates (1.5 × 10^5^ cells/well) at the ratio of 90:10 for 24 h, which were exposed to BPA or/and DIBP for 24 h. Subsequently, the absorbance at 450 nm was measured by Thermo Multiskan FC (Thermo Fisher Scientific, Waltham, MA, USA) according to the kit instructions. The effect of RHPE on cytotoxicity was determined using the same method as above.

### 2.10. Monolayer Integrity

Caco-2/HT29-MTX cells were seeded into the transwell chambers (12 wells, 0.4 μm) at a density of 2.25× 10^5^ cells/well. Refer to the previous cultural method [40]. Until the cells have fully differentiated after 21 days. RHPE treatment completely differentiated co-cultural cells for 24 h followed by BPA and DIBP for 24 h. Cells were grouped as follows: the control group (Con); BPA + DIBP (BD); BPA + DIBP + BBR50 (BBR); BPA + DIBP + RHPE50 (RHPE50); BPA + DIBP + RHPE100 (RHPE100); BPA + DIBP + RHPE150 (RHPE150) (Figure 1B).

After the electrode was activated, the TEER of the cells at three wells was measured and averaged. TEER was measured using Millicell^®^ ERS voltammeter (Millipore, Bedford, MA, USA). FD-4 was employed as a paracellular transport marker in Caco-2/HT29-MTX cells model. The apical chamber is 0.1 mg/mL FD-4, and the basal chamber is PBS. After incubating for 4 h in 37 °C, 5% CO_2_, take an appropriate amount of the lower layer solution and measure it at the excitation/emission wavelength of 490/520 nm by Thermo Multiskan FC (Thermo Fisher Scientific, USA).

### 2.11. Western Blotting

After the cells are fully lysed in the lysate, the protein is extracted for quantification. Then, the SDS-PAGE experiment was carried out, after transferring the protein onto PVDF membranes (0.2 μm pore, Millipore, St. Louis, MI, USA), which were then blocked in 1% BSA for 50 min at room temperature. Incubated with the corresponding primary antibody working solution overnight at 4 °C, and incubated with secondary antibody working solution for 50 min. Visualize protein bands using ChemiDoc Imaging Systems (Bio-Rad Gel Imaging Systems, Hercules, CA, USA) and quantitatively analyze by image J.

### 2.12. Statistical Analysis

SPSS 26.0 (SPSS Inc., Chicago, IL, USA) was employed for one-way ANOVA, with Duncan’s or Tamhane’s T2 method for multiple comparisons. The data were expressed as mean ± SD. There is a significant difference in value without a common superscript (*p* < 0.05). Furthermore, we conducted principal coordinate analysis (PCoA) and non-metric multidimensional scaling plot (NMDS) analyses of gut microbes.

## 3. Results

### 3.1. Effects of RHPE Interventions on Body Weight and Organ Index

**Figure 2:**
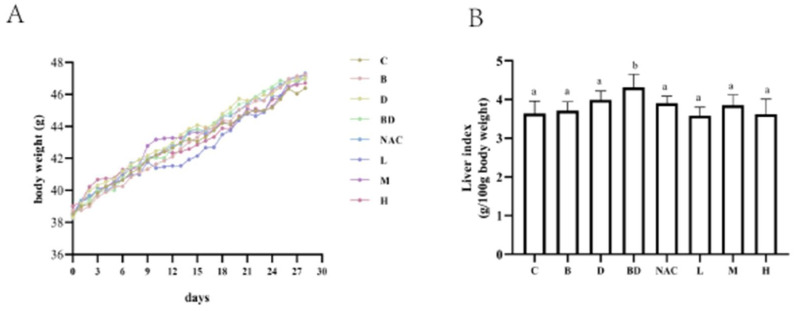
Effect of RHPE on organ index in mice. (**A**) Body weight change of the mice. (**B**) Liver index. Different letters indicated significant differences among groups (*p* < 0.05).

We used BPA combined with DIBP to establish an intestinal injury model in KM mice and administered different doses of RHPE intervention (Figure 1A). The body weight in each treatment group did not differ significantly (Figure 2A). The liver index increased significantly only in the BPA + DIBP group (*p* < 0.05) compared with the control group (Figure 2B). It was observed that RHPE pretreatment helped to reduce the liver indices of mice to a level that was comparable to that of the control group. However, groups B and D did not cause any significant harm to the livers of the mice.

### 3.2. Effects of RHPE Interventions on Intestinal Histopathology

**Figure 3:**
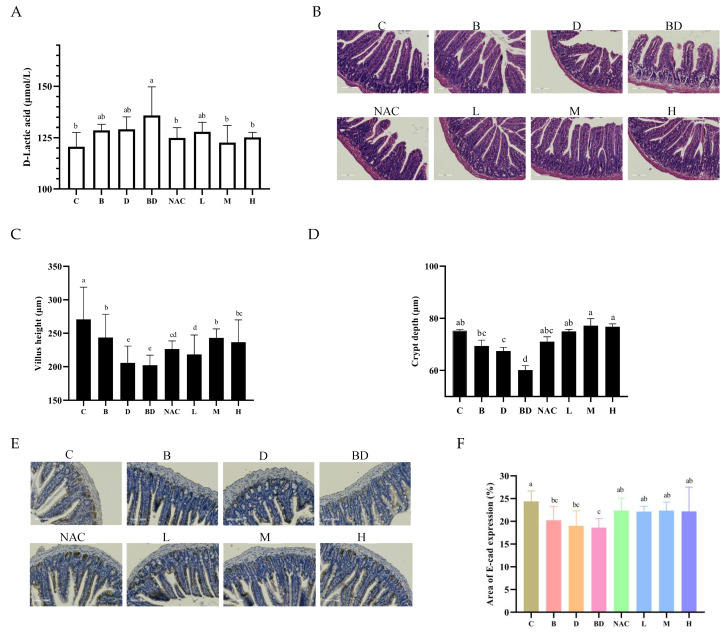
RHPE improved the pathological damage caused by BPA + DIBP combined exposure in mice. (**A**) Serum D-Lac levels in mice. (**B**) Representative images of H&E staining of ileum sections. (**C**,**D**) Villus height and crypt depth. (**E**,**F**) E-cad immunohistochemical staining analysis. Different letters indicated significant differences among groups (*p* < 0.05).

D-Lac enters the blood when the intestine barrier is damaged, causing a rise in D-Lac levels in the blood. As a result, D-Lac is frequently utilized as an indicator of intestinal injury [41]. Compared with the control group (121 ± 7 μmol/L), the level of D-Lac in the BPA + DIBP group (136 ± 14 μmol/L) was significantly higher (*p* < 0.05), and there were no notable changes in the B and D groups. This suggests that BPA and DIBP exposure alone did not result in significant harm to mice. However, when the two were combined, the intestinal permeability of mice was significantly increased. The serum D-Lac levels of mice exposed to combined BPA + DIBP were reduced under the RHPE intervention (Figure 3A).

The H&E staining results revealed the overall extent of the damage (Figure 3B). As shown in Figure 3C,D, villus height and crypt depth were significantly reduced in the BPA + DIBP group. This suggests that the combined exposure to BPA and DIBP may result in some degree of damage to the intestinal barrier. It is noteworthy that the intestinal damage induced by combined BPA and DIBP exposure was significantly reversed by RHPE. E-cadherin (E-cad) can span cell membranes, enhancing intercellular adhesion [42]. The combined effect of BPA + DIBP decreased the expression of E-cad in the tissues, and the E-cad level partially recovered after pretreatment with RHPE (Figure 3E,F). Thus, RHPE could inhibit histopathology injury induced by BPA and DIB.

### 3.3. RHPE Facilitated the Proliferation and Differentiation of ISCs

**Figure 4:**
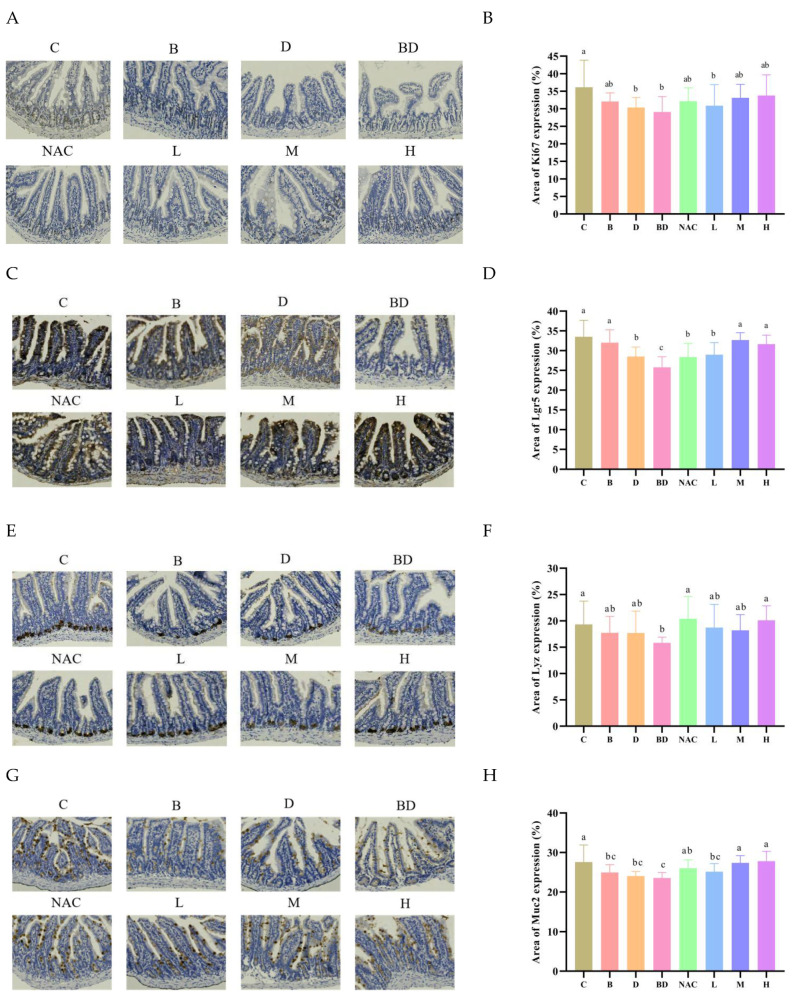

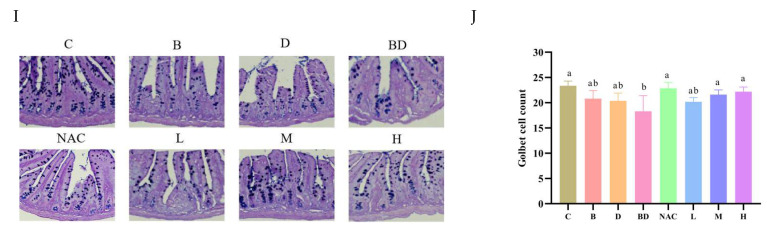
RHPE promotes the proliferation and differentiation of mouse intestinal stem cells. Immunohisto-chemical staining and relative positive area of (**A**,**B**) Ki67. (**C**,**D**) Lgr5. (**E**,**F**) Lyz. (**G**,**H**) Muc2. (**I**,**J**) AB–PAS staining of goblet cells. Different letters indicated significant differences among groups (*p* < 0.05).

Accumulated evidence suggests that the regeneration of the intestinal epithelium is crucial for maintaining intestinal barrier function. The expression level of Ki67 reflects the rate of cell proliferation; therefore, Ki67 was utilized to assess intestinal epithelial repair and renewal. We employed IHC staining to measure the expression of Ki67 in the mouse ileum (Figure 4A,B). Our data indicated a significant suppression of Ki67 expression in mice treated with BPA + DIBP, which was restored by RHPE pretreatment. And the expression of Lgr5 (a mature ISCs marker) in the ileum crypt. Lgr5 levels were significantly reduced under the BPA + DIBP treatment compared to the control group, and recovered after RHPE pretreatment (Figure 4C,D).

Moreover, ISCs can differentiate into functional enterocytes, such as goblet cells, Paneth cells, and enteroendocrine cells. We evaluated the differentiation of ISCs by measuring changes of Lyz (a marker of Paneth cells) and Muc2 (a marker of goblet cells). The results indicated that the combined action of BPA + DIBP could significantly affect the generation of Paneth cells and goblet cells. The RHPE intervention resulted in an increase in the region of positive Muc2 expression and no change in Lyz expression (Figure 4E–H). The mucus barrier plays an important role in maintaining intestinal homeostasis [43]. We further investigated the effect of RHPE on the number of cupped cells by AB–PAS staining. As depicted in Figure 4I,J, the BPA + DIBP group had considerably fewer goblet cells than the control group. Nevertheless, supplements with RHPE substantially improved the depletion of goblet cells induced by BPA and DIBP. To summarize, we employed the Caco-2/HT29-MTX co-culture model to validate the in vivo results.

### 3.4. RHPE Protected Against BPA- and DIBP-Induced Intestinal Injury In Vitro

**Figure 5:**
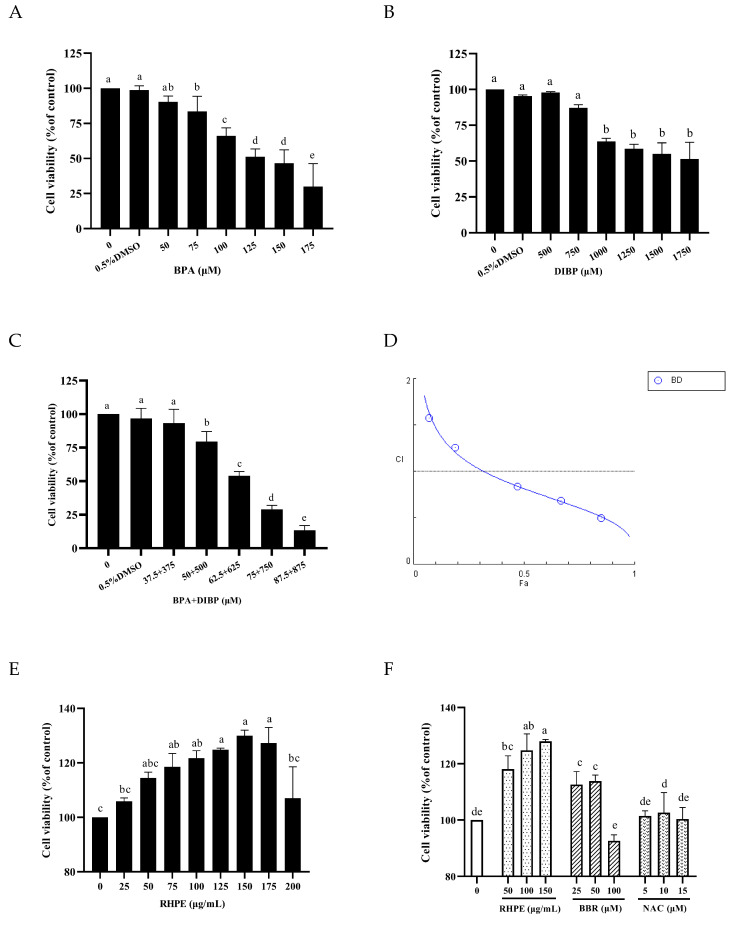
Caco-2/HT29-MTX Co-culture cytotoxicity of BPA, DIBP and RHPE. (**A**) BPA cell viability (24 h). (**B**) DIBP cell viability (24 h). (**C**) BPA + DIBP cell viability (24 h). (**D**) BPA and DIBP CI index analysis. (**E**) Effect of RHPE on cell viability. (**F**) BBR and NAC cell viability. Different letters indicated significant differences among groups (*p* < 0.05).

The experimental results indicated that the cell survival rate decreased as the dosage concentration increased (*p* < 0.05). The 24 h IC_50_ values of BPA and DIBP are approximately 125 μM and 1000 μM (Figure 5A,B). We conducted joint administration experiments at the ratio of BPA:DIBP = 1:10. When the concentration of BPA + DIBP was 62.5 μM + 625 μM, the cell viability of Caco-2/HT29-MTX cells was reduced to 54% (Figure 5C). Based on the CI index curve of the BPA and DIBP combination, it can be concluded that when the inhibition rate is >35%, the theoretical CI values corresponding to different doses are all <1, suggesting a synergistic effect between the two (Figure 5D).

Similarly, when the RHPE concentration is 125–175 μg/mL, the cell survival rate is the highest (Figure 5E). Thus, 50, 100, and 150 μg/mL for subsequent experiments. Cell viability was determined for NAC and Berberine (BBR) in the same manner as for RHPE; NAC pretreatment did not improve Caco-2/HT29-MTX co-culture model cell viability, however, BBR improved cell viability. BBR is a potent alkaloid extract derived from Phyllodendron Bark, boasting potent antimicrobial and antidiarrheal properties [44]. It is utilized clinically for the treatment of diarrhea and gastroenteritis [45]. Available research indicates that BBR enhances the barrier [46]. Therefore, 50 μM BBR was used as a positive control (Figure 5F).

### 3.5. RHPE Improved Cell Barrier Integrity and Permeability of Caco-2/HT29-MTX

**Figure 6:**
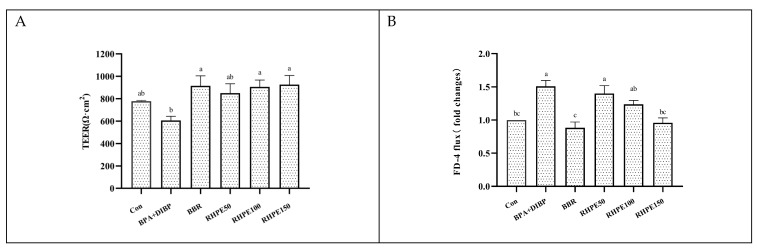
RHPE improved monolayer integrity and permeability of Caco-2/HT29-MTX co-cultured cells. (**A**) Monolayer integrity. (**B**) Permeability. Different letters indicated significant differences among groups (*p* < 0.05).

The cells are grouped as indicated in Figure 1B. Compared to the control group, the combined exposure to BPA + DIBP resulted in a remarkable reduction in the TEER value of Caco-2/HT29-MTX cells (*p* < 0.05). Nevertheless, RHPE pretreatment was found to significantly mitigate this trend (Figure 6A). In addition, the FD-4 transmittance in the BPA + DIBP group was significantly increased in comparison to the control group (*p* < 0.05), and the cell monolayer transmittance was reduced after the addition of RHPE (Figure 6B). Taking these results together, RHPE could mitigate the reduction in cellular barrier integrity induced by BPA and DIBP.

### 3.6. RHPE Alleviated Goblet Cell Damage Through Notch Pathway

**Figure 7:**
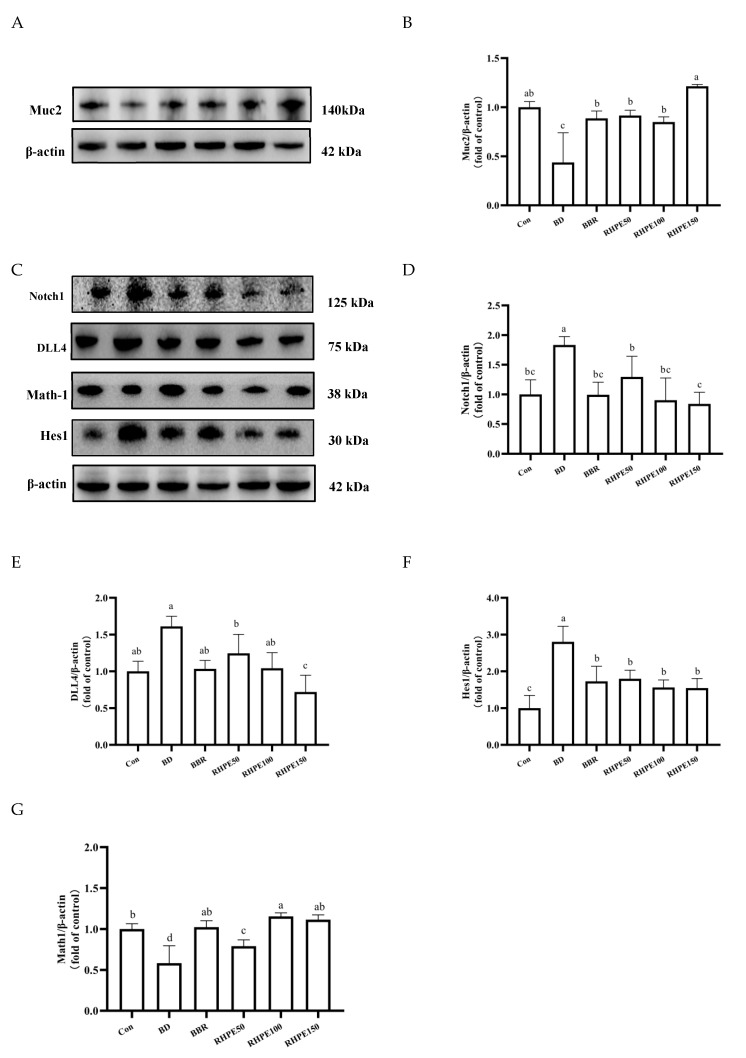
RHPE alleviates goblet cell damage through DLL4–Notch1–Hes1–Math1 pathway. (**A**,**B**) Relative protein expression of Muc2. (**C**) Notch pathway key protein expression was measured by Western blotting in Caco-2/HT29-MTX. (**D**–**G**) Notch1, DLL4, Hes1, Math1. Different letters indicated significant differences among groups (*p* < 0.05).

Glycosylated Muc2 is the core of mucus. Our data indicated a reduction in the content of Muc2 in the presence of the BPA + DIBP group, with a corresponding increase in the production of Muc2 in the RHPE50, RHPE100, and RHPE150 groups. These results demonstrated that RHPE plays a vital role in stimulating the expression of Muc2, therefore, regulating mucus secretion (Figure 7A,B). The concept of mucin secretion by goblet cells is well established; however, the impact of BPA and DIBP on Muc2 secretion through the Notch pathway remains unclear. We quantified the expression levels of DLL4, Notch1, Hes1, and Math1, the key proteins of the Notch signaling pathway by Western blot. We aim to determine if RHPE intervention plays a role in promoting the differentiation of ISCs into goblet cells (Figure 7C). The Notch pathway was significantly activated in the BPA + DIBP group by the rise in the expression level of the target gene Hes1. Nevertheless, RHPE intervention was able to mitigate the up-regulation of Hes1 compared to the BPA + DIBP group. In accordance with expectations, the level of Math1, a transcription factor repressed by Hes1, decreased after BPA + DIBP treatment. Interestingly, Math1 was restored by RHPE supplementation, thereby hastening the growth of goblet cells (Figure 7D–G). All of these results suggested that RHPE regulates the overactivated Notch pathway, stimulating Muc2 secretion and enhancing the mucus barrier.

### 3.7. Effect of RHPE on Gut Microbiota

**Figure 8:**
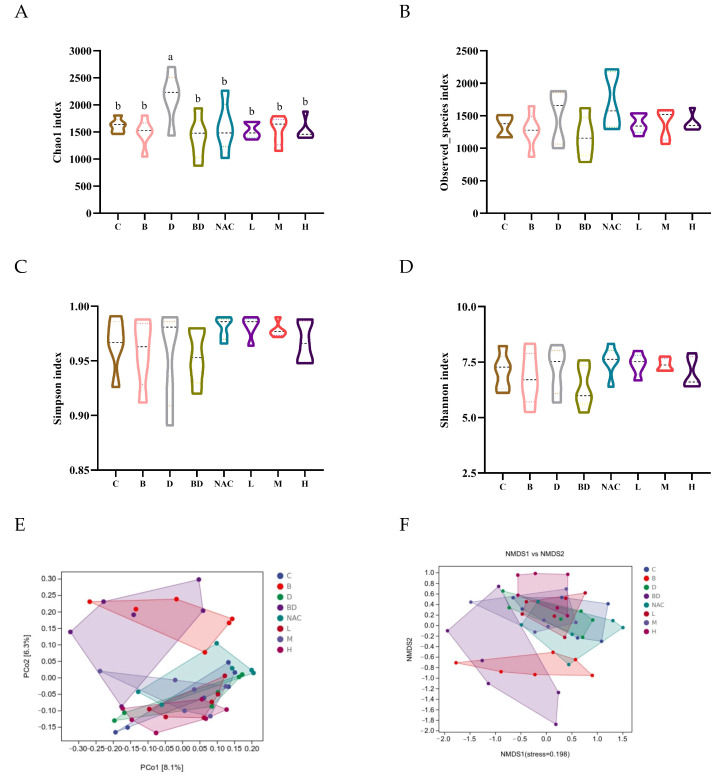
Effects of RHPE on gut microbiota in BPA- and DIBP-induced mice. (**A**) Chao1 index. (**B**) Observed-species index. (**C**) Simpson index. (**D**) Shannon index. (**E**,**F**) Principal coordinate analysis (PcoA) and NMDS of β diversity. Different letters indicated significant differences among groups (*p* < 0.05)

We analyzed the species composition and diversity of the colonic contents to determine the effect of RHPE on the gut microbiota of mice exposed to BPA and DIBP. The richness of the microbial community is shown by Chao1 and Observed species indices, while the Simpson and Shannon indices show the community diversity [47]. Compared to the control group, the BPA + DIBP group showed a significant decrease in the Chao1 indices. This implies that RHPE has the potential to enhance the abundance of the gut microbiota, whereas it has no significant impact on the diversity of the gut microbiota (Figure 8A–D). Furthermore, we used PCoA and NMDS to assess the Beta diversity. The results showed that RHPE can restore the intestinal flora structure in mice that have been exposed to a combination of BPA and DIBP, as well as match the composition of the microbial community to that of the mice in the control group (Figure 8E,F).

**Figure 9:**
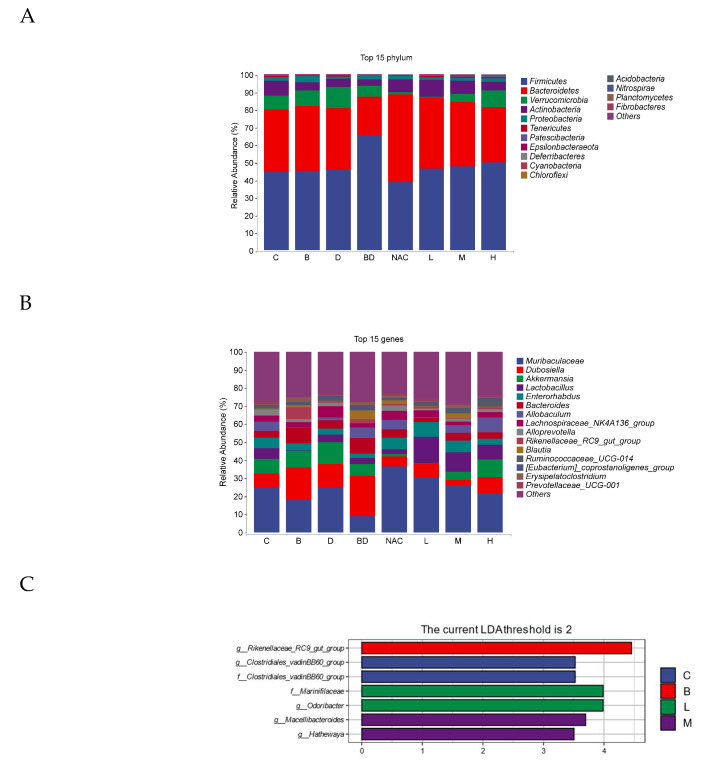
Effects of RHPE on intestinal microbial disorders in mice induced by combined exposure to BPA and DIBP. (**A**) Taxonomic composition at phylum level. (**B**) Taxonomic composition at genus level. (**C**) LDA analysis.

At the phylum level, the gut microbiota primarily consists of Firmicutes, *Bacteroidetes*, *Verrucomicrobia*, and *Actinobacteria*. Compared with the control group, the populations of *Firmicutes* were significantly increased, whereas the abundance of *Bacteroidetes*, *Verrucomicrobia*, and *Actinobacteria* was markedly decreased in the BPA + DIBP group (*p* < 0.05). In contrast, compared with the BPA + DIBP group, RHPE treatment resulted in lower *Firmicutes* and a higher *Bacteroidetes*, *Verrucomicrobia*, and *Actinobacteria* (Figure 9A).

The composition of intestinal flora at the genus level is shown in Figure 9B. At the gene level, the sample was dominated by *Muribaculaceae*, *Dubosiella*, *Akkermansia*, and *Lactobacillus*. *Muribaculaceae*, *Akkermansia*, and *Lactobacillus* abundance was substantially lower while the populations of *Dubosiella* were significantly higher in the BPA + DIBP group compared with the control group. RHPE intervention effectively normalized imbalanced gut microbiota.

In order to determine the bacteria alteration by the RHPE administration, biomarkers between several groups were identified using linear discriminant analysis (LDA). Data indicated that the control group was significantly enriched in both the g_Clostridiales_vadinBB60_group and the f_Clostridiales_vadinBB60_group. The BPA group was enriched considerably in the g_Rikenellaceae_RC9_gut_group, while group L was significantly enriched in f_Marinifilaceae and g__Odoribacter, and group M was significantly enriched in g_Macellibacter oides and g__Hathewaya (Figure 9C).

### 3.8. RHPE Improved SCFAs Abundance

**Figure 10:**
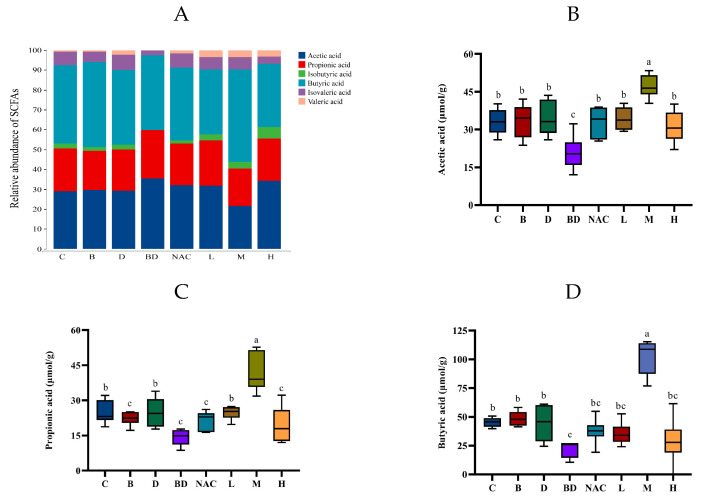
Effects of RHPE on the content of SCFAs in mice. (**A**) Relative content of SCFAs. (**B**) Acetic acid. (**C**) Propionic acid. (**D**) Butyric acid. Different letters indicated significant differences among groups (*p* < 0.05).

We measured the impact of RHPE on SCFAs (metabolites of the intestinal flora) using the GC system, and determined the concentrations of acetic, propionic, isobutyric, butyric, isovaleric, and valeric acids in the colonic contents. As depicted in Figure 10A, acetic acid, propionic acid, and butyric acid were the predominant SCFAs present in the intestinal contents. The BPA + DIBP group showed significantly lower levels of acetic acid, propionic acid, and isobutyric acid compared to the control group. After pretreatment with 400 mg/kg RHPE, the levels of acetic acid, propionic acid, and isobutyric acid were significantly restored (Figure 10B–D). It is evident that RHPE intervention had a substantial improvement effect.

## 4. Discussion

Plasticizers can be absorbed into the human body through the digestive tract, respiratory tract, and skin. The majority of current studies of plasticizers are based on a single exposure. Nevertheless, people are often exposed to a mixture of two or more plasticizers in their everyday lives. Plasticizers have various toxic effects, including renal toxicity, endocrine toxicity, reproductive toxicity and so on. Studies have indicated that natural antioxidants, such as polyphenols, possess antimicrobial activity against common foodborne pathogens as well as antioxidant, hypoglycemic and hypolipidemic effects. Based on this scenario, RHPE has caught our attention. Therefore, this study investigated the protective effect of RHPE against BPA- and DIBP-induced intestinal damage.

BPA and DIBP exposure were found to significantly alter the composition of the intestinal flora in mice. Then, changes in the microbiota result in modifications to its derived metabolites, which have a significant impact on intestinal health [48]. Hence, maintaining the homeostasis of the intestinal flora is a promising approach to ameliorating the effects of plasticizers on intestinal damage. Notably, the intestinal absorption of RHPE is poor, with the majority of it remaining within the intestinal lumen where it interacts directly with microorganisms. As a result, the gut flora may be a crucial target for the RHPE to exert a therapeutic effect, as our recent experiments have confirmed.

The disruption of intestinal barrier integrity may occur under the influence of combined BPA and DIBP exposure, leading to the infiltration of harmful bacteria into the intestinal lumen. In a study that preliminarily demonstrates that exposure to PAEs can alter the gut microbiota of adolescent rats, the abundance of *Bacteroides* in the gut increased and that of *Firmicutes* decreased after 30 days of consecutive exposure to microplastics (MPs) and PAEs [49]. In our study, the ratio of F/B was significantly higher in the BPA + DIBP group compared to the control group; however, the addition of RHPE (200, 400, 600 mg/kg) significantly reduced the F/B level. Similarly, after 8 weeks of administration at a dose of 200 mg/kg, a grape seed proanthocyanidin treatment normalized the F/B ratio in C57BL/6J mice, reversing the HFD-induced reduction in the relative abundance of *Akkermansia* [50]. *Muribaculaceae* is a beneficial bacterium found in the gut microbiota of mice, which contributes to preventing intestinal barrier dysfunction, inflammation, and lipid metabolism disorders [51]. The pretreatment of RHPE mitigates the reduction in *Muribaculaceae* abundance induced by the combination of BPA + DIBP. Mucus was present on the outer layer of epithelial cells and served to shield the epithelial cells from harmful microorganisms that could enter the intestinal lumen. *Akkermansia* promotes the growth and maintenance of the intestinal mucus layer and improves the barrier function of the intestine [52]. Wu demonstrated that oral administration of Epigallocatechin-3-gallate (EGCG) at a dose of 50 mg/kg for 3 consecutive days significantly increased the abundance of beneficial bacteria such as *Akkermansia* and the content of SCFAs in mice that were subjected to DSS-induced colitis [35]. *Lactobacillus* is believed to enhance intestinal barrier defenses by promoting mucus secretion and adhesion to mucin and intestinal epithelial cells [53]. Our study showed that the RHPE pretreatment increased the relative abundance of intestinal flora, especially, *Akkermansia* and *Lactobacillus*. *In vitro* studies indicated that 10^10^ colony-forming units (CFUs)/mL of *Lactobacillus plantarum* PS128 was capable of promoting intestinal motility and mucin production [54]. *L rhamnosus* CNCM I-3690 protected or restored goblet cell populations and reduced the thickness of the mucus layer in mice after low-grade colitis. This is in line with our study, where dietary supplementation with RHPE maintained the *Akkermansia* and *Lactobacillus* barrier function of the intestine. The above findings indicate that RHPE regulates the intestinal barrier and maintains intestinal homeostasis through alterations in the composition structure and diversity of the gut microbiota.

The function and microenvironment of the large intestine differ from that of the small intestine, which is more susceptible to bacterial attack. Thus, the integrity of the mucosal barrier is crucial for the small intestine [55]. Combined exposure to BPA and DIBP increased serum D-Lac levels and increased intestinal permeability. After the RHPE pretreatment, the damage to intestinal villi and the serum D-Lac concentration was dramatically ameliorated. By intragastric administration of RHPE in mice, pathological results also showed that RHPE significantly improved intestinal barrier damage caused by BPA + DIBP. The intestinal barrier consists of different intestinal epithelial cells (such as goblet cells, Paneth cells, and enteroendocrine cells) that are produced from intestinal stem cells. It is a constantly regenerating single cell layer. We hypothesize that intestinal stem cell differentiation and proliferation play a role in the intestinal barrier repair process caused by RHPE. Our examination of the staining of various intestinal cells revealed that RHPE mainly facilitated the differentiation of ISCs into goblet cells and restored the decreased expression of Muc2. Similar to our results, aloe vera gel (200, 400 mg/kg) was shown to increase the number of ISCs, promote differentiation of ISCs into intestinal epithelial cells, and repair damaged intestinal epithelium [56]. This evidence suggests that RHPE can regulate the renewal and proliferation of ISCs, which in turn leads to the improvement of intestinal barrier function.

The continuous production and secretion of mucin by goblet cells is crucial for maintaining the protective lining of the intestine. Mucus barrier disruption is one of the causes of intestinal damage [57]. According to reports, animals lacking Muc2 are more susceptible to bacterial invasion, leading to intestinal inflammation and possibly spontaneous colitis development [58]. In the present study, the combination of exposure to BPA and DIBP resulted in goblet cell injury and a decrease in Mcu2 secretion in mice. However, in vivo research shows that RHPE reduces intestinal damage by encouraging intestinal stem cells to differentiate into goblet cells, increasing the quantity of goblet cells, and ultimately boosting mucin secretion. In order to test this conclusion, we used the Caco-2/HT29-MTX co-culture model as the experimental object to confirm the impact of RHPE on goblet cells and investigate the mechanism of RHPE. Interestingly, the survival rate of Caco-2/HT29-MTX cells was significantly improved under RHPE treatment. Further experiments showed that RHPE prevented intestinal barrier damage caused by combined exposure to BPA and DIBP. Additionally, the therapeutic effect of RHPE on intestinal mucosal damage in mice caused by BPA and DIBP may be related to enhancing the production and secretion of Muc2. However, the production and secretion of Muc2 may be related to inhibiting the Notch signaling pathway.

In brief, the Notch pathway is a crucial pathway that regulates the proliferation and differentiation of ISCs [59,60]. The activation of the Notch pathway is dependent on the interaction between receptors and ligands in neighboring cells. Notch protein is cleaved into NICD and released into the cytoplasm, which enters the nucleus to form a transcriptional activation complex NICD/CSL). This complex leads to the transcriptional activation of Hes1 (a Notch target gene), which inhibits the expression of transcription factor Math1 [61]. Further restricts the differentiation of ISCs into goblet cells, resulting in reduced mucus secretion. It was reported that the Notch pathway is overactivated in patients with colitis, Aloin A prevents ulcerative colitis in mice by enhancing the intestinal barrier function via suppressing the Notch pathway [57]. Our findings demonstrate that RHPE can suppress the high activated Notch signaling pathway and enhance the mucus barrier. Similarly, research has shown that Bacillus coagulans can improve goblet cell loss and decrease Muc2 expression caused by Salmonella enteritis [61].

## 5. Conclusions

Our findings demonstrate that exposure to BPA and DIBP can result in intestinal mucosal barrier damage and that the combination of the two has exacerbated the toxic effects in mice. It is noteworthy that the administration of RHPE partially alleviated the damage caused by the combined exposure of BPA and DIBP in mice. RHPE has a modulatory effect on the Notch pathway and the gut flora, exerting health benefits in preventing human intestinal damage. Subsequently, we will investigate the precise mechanisms by which RHPE impacts the intestinal mucosal barrier through the gut flora and its metabolites through the use of flora transplantation and molecular docking.

## Figures and Tables

**Table 1:** Mouse administration schedule.

Group	Days 1–7	Days 8–28	
		8:30 a.m.	10:30 a.m.
C	Normal saline	Corn oil	Corn oil
B		Corn oil	BPA
D		Corn oil	DIBP
BD		Corn oil	BPA + DIBP
NAC		NAC	BPA + DIBP
L		RHPE200	BPA + DIBP
M		RHPE400	BPA + DIBP
H		RHPE600	BPA + DIBP

## Data Availability

The original contributions presented in the study are included in the article, and further inquiries can be directed to the corresponding author.

## Abbreviations

BPA: bisphenol A; DIBP, diisobutyl phthalate; RHPE, *Rubus chingii* Hu phenolic extract; D-Lac, D-lactic acid; H&E, hematoxylin and eosin; AB–PAS, alcian blue and periodic acid schiff; IHC, immunohistochemical; ISCs, intestinal stem cells; SCFAs, short-chain fatty acids; CI, combination index; TEER, transepithelial electrical resistance; FD-4, fluorescein isothiocyanate dextran 4.
